# The Lords' Committee

**Published:** 1891-03-14

**Authors:** 


### The Lords' Committee.

On March 5th the Royal Free Hospital was the subject of inquiry, and the noble Lords betrayed much interest in details concerning the women medical students who walk the wards there. Mr. O. W. Thies, examined by the Chairman, stated that he was, and had been for three years, Secretary of the Royal Free Hospital in Gray's Inn Road. This was an entirely free hospital, and no governor's letters were issued. There was a system of selection by the responsible medical officers, and letters of admission. The government of the hospital was entrusted to a court of governors, a committee of management, and the weekly board. He was supreme in the absence of the board, and he was responsible to them. There was a total of 160 beds, and the working average was 135. All except infectious cases were taken in. Every week patients had to be turned away because there was not room for them. Mrs. Garrett-Anderson, the Dean of the Ladies' Medical School attached to the hospital, was next examined, and, in reply to the Chairman, she stated that the ladies paid fees of £110, of which £70 went to the school and the rest to the hospital. There were 107 students, and the number had been increasing of late years. Last year's entries were the largest in number. There were thirty-four new students at the beginning of October. That was the only hospital in England where ladies could study medicine, and it was the only medical ladies' school in England. There were about 115 to 120 duly qualified medical women practitioners on the register, and most of them were in practice, many of them abroad. She thought it a very good profession. Perhaps women were not so ambitious as men were, but they had reason to be satisfied. She sent a student out to Queensland this week for a good appointment. Women had several times been appointed house surgeons at Birmingham, and she thought a lady at Bristol had some sort of hospital appointment. Mr. Alderson, a general practitioner, then gave evidence to the effect that he considered the hospitals abused, and thus injury was done to the private practitioner. Lord Cathcart said he thought the evil arose because the medical profession was over-stocked.

On Monday last Mrs. Alison gave evidence. She desired to contradict a statement made by the Sister Cecilia, Lady Superintendent of the nursing establishment of University College Hospital. She stated that it was not correct to say that no distinction in the selection of probationers was made on account of their religious persuasion. Witness was a Presbyterian, and when that fact became known, her application to be taken on was declined. She was told that she was ineligible. She had made no representation on the subject to the hospital authorities, as she was under the belief that the power of exclusion rested with the Matron. Mr. C. H. Byers, Secretary of the Metropolitan Hospital, Kingsland Road, was next examined with reference to the provident system which prevailed at the hospital. He stated that the urgent cases were perfectly free. The provident department was intended for persons unable to pay for medical advice. A single person who earned not more than 21s. a week or a married person whose family earned not more than 35s. a-week, could become a member of the department. There was a system of regular payment of a penny a-week for adults and twopence a-month for children, but sixpence a-month would include all the children of a family under sixteen. Contributors did not receive benefits until they had made two months' payment. The number of new cases were 14,000 last year, and the total attendance was 23,000. The amount received in provident payments last year was £670 4s. 7d., and the previous year £653. The Jews had been very liberal towards the hospital, and it had been resolved that out of 160 beds 40 should be left for persons of the Jewish persuasion, and there were separate kitchens for the cooking of their food. The witness was then examined as to the constitution of the governing body and the nursing arrangements. A letter was read from Sister Cecilia, stating that Mrs. Alison's application must have been made before it was decided, in 1889, to admit nurses of all creeds.

